# An adaptive hybrid XdeepFM based deep Interest network model for click-through rate prediction system

**DOI:** 10.7717/peerj-cs.716

**Published:** 2021-09-17

**Authors:** Qiao Lu, Silin Li, Tuo Yang, Chenheng Xu

**Affiliations:** 1 Taicu Music co Ltd Shenzhen China, Shenzhen, United Kingdom; 2 School of Economics, Tianjin University of Commerce, Tianjin, China

**Keywords:** Click-Through Rate Prediction, Hybrid model, Deep Interest Network, XDeepFM, Parallel ensemble, OHEM, Neural Networks, Deep Learning, Machine Learning

## Abstract

Recent advances in communication enable individuals to use phones and computers to access information on the web. E-commerce has seen rapid development, e.g., Alibaba has nearly 12 hundred million customers in China. Click-Through Rate (CTR) forecasting is a primary task in the e-commerce advertisement system. From the traditional Logistic Regression algorithm to the latest popular deep neural network methods that follow a similar embedding and MLP, several algorithms are used to predict CTR. This research proposes a hybrid model combining the Deep Interest Network (DIN) and eXtreme Deep Factorization Machine (xDeepFM) to perform CTR prediction robustly. The cores of DIN and xDeepFM are attention and feature cross, respectively. DIN follows an adaptive local activation unit that incorporates the attention mechanism to adaptively learn user interest from historical behaviors related to specific advertisements. xDeepFM further includes a critical part, a Compressed Interactions Network (CIN), aiming to generate feature interactions at a vectorwise level implicitly. Furthermore, a CIN, plain DNN, and a linear part are combined into one unified model to form xDeepFM. The proposed end-to-end hybrid model is a parallel ensemble of models via multilayer perceptron. CIN and xDeepFM are trained in parallel, and their output is fed into a multilayer perceptron. We used the e-commerce Alibaba dataset with the focal loss as the loss function for experimental evaluation through online complex example mining (OHEM) in the training process. The experimental result indicates that the proposed hybrid model has better performance than other models.

## Introduction

Nowadays, thanks to communication technologies, the Internet has connected people worldwide, and web and mobile applications have been applied widely and become indispensable in most countries (Bahdanau, Cho & Bengio, 2014). Increasingly, people tend to learn or consume on the Internet, producing many web browsing behaviors. Based on these, some enterprises place ads on a variety of pages to engage customers. The suitable ads for the query and the order in which they are displayed are significant for enterprises to increase their revenue from ads and essential for users to improve their experience.

CTR (Richardson, Dominowska & Ragno, 2007) indicates the number of ad clicks an advertiser receives per impression. CTR plays an essential role in the Pay-Per-Click (PPC) advertising model, an online advertising model that indicates advertisers’ payment each time a user clicks on an online ad. In a Cost-Per-Click (CPC) advertising system such as Alibaba (Mangani, 2004; Hu, Shin & Tang, 2016), the cost of advertising is based on the effective cost per thousand people (eCPM), the price of the advertised product, and CTR. Artificial Intelligence (AI) (Albahli et al., 2021), including subareas such as deep learning (Gao, Wang & Shen, 2020c; Gheisari et al., 2021), machine learning (Gao, Wang & Shen, 2020a; Gao, Wang & Shen, 2020b), and neural networks (Rauf et al., 2018), plays an essential role in applying prediction and forecasting to e-commerce (Liang et al., 2021; Wei et al., 2020), Healthcare (Meraj et al., 2019), Optimization (Rauf, Bangyal & Lali, 2021; Rauf et al., 2020), and IoT industry applications (Malik et al., 2021).

**Figure 1:**
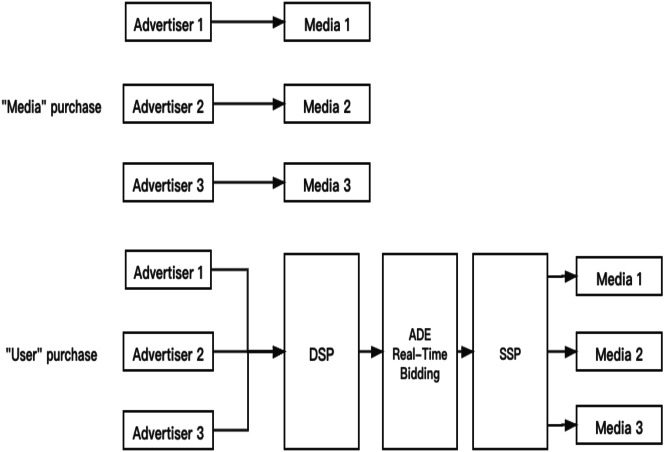
Advertisement CTR prediction system.

Business Intelligence has received much attention in the last decade, in which CTR prediction is a critical metric in the recommended system. For e-business recommendations (Purushotham, Liu & Kuo, 2012), it needs to predict CTR and CVR(the user’s conversion rate). For content recommendation (Davidson et al., 2010), the business side requires reading time, comments and CTR, etc. Advertisers purchase keywords covering services or products (Amodei et al., 2016; Ji et al., 2019). When users retrieve these keywords through the Internet, the website will automatically display relevant advertisements to users. When the user clicks on the ad or visits the relevant advertisement page, the website can collect payment from the advertiser. Therefore, the performance of the CTR prediction model directly affects the final income of the enterprise. The system of the advertisement system is shown in Fig. 1, and it has three kinds of purchases. They are “Media” purchase, “Crowd” purchase, and “User” purchase.

This study developed a hybrid model integrating the Deep Interest Network (DIN) and xDeepFM. DIN uses an adaptive local activation unit that includes an attention mechanism to learn user interest from previous behaviors related to adaptively specific advertisements. CIN, a fundamental component of XDeepFM, aims to produce feature interactions at a vectorwise level automatically. In addition, xDeepFM combines a CIN, a plain DNN, and a linear component into a single unified model. The proposed end-to-end hybrid model is a multilayer perceptron-based parallel ensemble of models. The output of CIN and xDeepFM is input into a multilayer perceptron, which is trained concurrently. For experimental evaluation through OHEM in the training process, we employed the e-commerce Alibaba dataset with the focal loss as the loss function.

## Literature Review

This section will discuss some research and achievements of the advertisement CTR prediction system. Using only basic features does not generally provide optimal results, so both industry and academia attach great importance to transforming natural elements. There are two significant kinds of change (Guo et al., 2017). The first is constructing functions based on the multiple-feature combination (also called feature cross), using their output as the input to the learner; however, building cross-features manually comes with a high cost (Chen et al., 2016). The second is to use deep learning algorithms to implement feature learning without manual intervention (Duchi, Hazan & Singer, 2011). We will introduce some classic algorithms briefly. They are traditional algorithms, deep learning algorithms for CTR predictions, and composed algorithms.

### Traditional algorithms

The most classic statistical learning algorithm is Logistic Regression (Kumar et al., 2015), which has excellent characteristics such as simplicity, low time complexity, and large-scale parallelization. In the early CTR prediction period, algorithm engineers gave the nonlinear learning ability of the dataset to linear models such as the Logistic Regression model by manually designing cross-features and feature discretization. CTR prediction using Logistic Regression has become a baseline model to estimate a new model’s performance. Using this algorithm for CTR prediction, the predicted hypothesis in Logistic Regression will be evaluated as probabilities, always lying between 0 and 1. However, Logistic Regression relies on manual feature engineering, which has less efficient operations that require vast amounts of time. In a word, there are two primary characteristics of CTR prediction using a Logistic Regression algorithm: high-dimensional discrete features and manual cross-features.

As there are many drawbacks of Logistic Regression for CTR prediction, obtaining high-quality results is a costly endeavor. Correct features are usually task-specific (Chen et al., 2012), which means it is difficult to explore the potential pattern from the product data. Besides, as the advertising system is vast, extracting all cross-features from the raw data is a near-impossible task (Cheng et al., 2016). Manual feature engineering cannot be extended to invisible interactions in the training data. Therefore, autonomous learning of interactive features is essential and valuable work.

A new stage of feature engineering began in 2014. Facebook (Cho et al., 2014; Lian et al., 2017) proposed a CTR prediction model based on the GBDT (Lian et al., 2017) and the LR algorithm, which uses GBDT for automatic modeling and combination to reduce the complex manual feature engineering process. Due to the natural advantage of the tree model—combining high-order features and selecting appropriate features (preferring the split feature and split point with the most significant gain for each split)—the GBDT utilized in feature engineering is taken for granted. It is necessary to point out that the model is not an end-to-end model, which means feature transformation with the GBDT and LR parts are separate. The parameter of GBDT will not update when training the Logistic Regression part. There are three downsides of this hybrid model. It is a two-stage model, and the tree model is not applicable for handling massive high-dimensional sparse features. In addition, as the model is complex, it is difficult to deploy it online. Thus, this model is not sensitive to the data.

The Factorization Machine (FM) (Ling et al., 2017; Xiao et al., 2017) was proposed by Steffen Rendle in 2010 to solve feature combination problems under sparse data. Unlike the traditional simple linear model, the factorization machine considers the intersection between features and models all nested variable interactions (similar to the kernel function in SVM). In addition, the FM model can be calculated in linear time and integrated with many advanced collaborative filtering methods (such as Bias MF, svd++, etc.). For each original feature, FM will learn a hidden vector. The model automatically identifies effective feature combinations by enumerating all feature pairs and detecting the weights of feature pairs one by one. The importance of the feature pair is calculated by the inner product of the hidden vectors of the two original features involved in the feature pair. The advantage of FM lies in its handling of both feature combinations and the dimensionality guarantee. The first is feature combination. By combining the pairwise features, cross-term features are introduced to improve the model score; the second is to deal with the dimensional explosion by submitting hidden vectors (and matrix decomposition of the parameter matrix) to complete the parameter estimation of the features.

In a word, in the traditional CTR prediction field, there are linear models and nonlinear models. Linear models, such as the logistic regression model, learn efficiently and can be deployed quickly. However, their performance is not optimal because they cannot learn nontrivial patterns to catch interactions. On the contrary, nonlinear models can see the cross-feature, which improves estimation performance. However, the combination of features is difficult to exhaust, so these models cannot mine all possible combinations of different parts. In addition, another problem with the traditional click advertising rate modeling model is that because the model structure is relatively shallow, deep features cannot be extracted, and the expression ability is limited. The model cannot be modeled from a large amount of complex data; therefore, their data and generalization capabilities are limited.

### Deep learning algorithms

Microsoft pioneered deep learning on CTR prediction in 2016 (Yuan et al., 2016; Zhang et al., 2016). Deep Crossing extends deep understanding to a more general environment as deep knowledge is applied in both image and speech fields. It can receive unique features such as text, classification, ID, and numeric attributes and automatically search for the best combination according to various specific tasks using Deep Learning. Individual features have different properties. Therefore, determine how deep learning results in CTR estimation through feature learning representations on many rich domain discrete classification features. Deep Crossing covers the common elements of the deep CTR model: adding an embedding layer to convert sparse features into low-dimensional dense components; using a stacking layer to connect the segmented feature vectors; completing, through a multilayer neural network, the combination and conversion of features; and finally, using a scoring layer to complete the calculation of CTR. Unlike the traditional DNN, Deep Crossing’s multilayer perceptron comprises residual networks, profiting from the well-known 152-layer ResNet.

It is computationally expensive to train deep neural networks (DNNs) on an ample input feature space, requiring many parameters. The embedding layer of the FNN(Factorization-Machine-assisted Neural Network) (He & Chua, 2017; Kang et al., 2020) is a supervised-learning Factorization machine, which efficiently decreases the dimension from extra features to dense continuous elements, as opposed to the Deep Crossing. Using pre-trained training methods to complete the embedding layer is practical engineering training, which reduces the strength of the deep learning model and training instability.

In addition, the combination model has been exposed to getting better performance. The representative of this is Wide&Deep (Cheng et al., 2016; Matthews et al., 2018): an algorithm proposed by Google in 2016 for the Google Play app recommendation service. The core idea is to learn and model user behavior information by combining the memory of the Wide linear model and the generalization of the Deep depth model. The primary function of the Wide part is to make the model memorable. The single-layer Wide part can handle a large number of sparse id features so that the model can directly remember a large amount of historical information for the user. The primary function of the Deep part is to generalize the model and use the solid expressive ability of DNN to mine the data patterns hidden behind the features.

## Methodology

### Preliminary

#### Feature engineering

In the field of CTR prediction, the input feature dimensions are sparse, and there is no obvious spatial or temporal correlation (Shan et al., 2016; Xiao et al., 2017). The training dataset is shown as (*x*, *y*), where *X* mostly involves the information from user and item. Categorical fields and continuous fields are both included. Each classification field is shown as a one-hot encoding vector, and each continuous field is represented as a value itself or a discretized one-hot encoding vector. Each instance is converted to (*x*, *y*). One input instance [user id = c11, gender = female, date = Wednesday, interests = reading, ad id = book] is generally translated into high-dimension sparse features using encoding-field-aware one-hot encoding:

(1) \begin{eqnarray*}\underbrace{ \left\{ \left[ 0,\ldots ,1,\ldots ,0 \right] \right\} {}}_{ \left\{ user\text{_} \left\{ id \right\} =c11 \right\} },\underbrace{ \left\{ \left[ 0,1 \right] \right\} {}}_{ \left\{ gender=female \right\} },\underbrace{ \left\{ \left[ 0,0,1,0,0,0,0 \right] \right\} {}}_{ \left\{ date=Wednesday \right\} },\underbrace{ \left\{ \left[ 0,\ldots ,1\cdots \,,0 \right] \right\} {}}_{ \left\{ interest=study \right\} },\underbrace{ \left\{ \left[ 0,\ldots ,1,\ldots ,1,\ldots ,0 \right] \right\} {}}_{ \left\{ a{d}_{id}=book,~pencial \right\} }\nonumber\\\displaystyle \end{eqnarray*}

*y* ∈ 0, 1 means the associated label indicating user click behavior (*y* = 0 indicates that the user does not click the item, and *y* = 1 means otherwise). The mission of CTR prediction is to build a prediction model $\hat {y}=CTR.model(x)$ to calculate the likelihood of a user clicking on a specific app in a given situation.

#### Embedding layer

The embedding layer’s goal is to convert the high-dimensional binary vectors in the input into low-dimensional dense representations (Vo & Hays, 2019; Xu et al., 2017). The embedding layer is applied to the original function input, and the original data are compressed into a low-dimensional dense vector of actual values. If the field is not irresolvable, functional embedding is used as field embedding. The outcome of the embedding layer is a concatenated vector with the following format: *e* = [*e*1, *e*2, *dotsem*], where *einRD* signifies the embedding of one field. Different length instances can be translated into the same dimension *mtimesD*, where m denotes the number of fields and D denotes the field embedding dimension.

### Hybrid model

#### Deep interest network

When it comes to CTR prediction challenges, all deep learning systems follow the same paradigm: embedding and MLP. The large-scale sparse input features are first transferred to low-dimensional embedding vectors and, subsequently, fixed-length vectors. Finally, they are connected and fed into a fully connected layer (sometimes referred to as a multilayer perceptron or ML) to learn one of the nonlinear relationship’s properties.

From many experiments, we find that the bottleneck of expressing a user’s diverse interests in Embedding and MLP is the user representation vector with a limited dimension. Different users have diverse interests in the shopping scene, captured from users’ behavior data, which primarily affects CTR prediction. Nevertheless, in traditional methods using deep learning, nearly all of them learn to represent all user behaviors in a fixed-length vector. Unfortunately, significantly increasing the size of the learning parameters will raise the risk of overfitting with minimal data. Furthermore, it introduces the computational and storage overhead and is incompatible with online systems. Only a portion of the user’s interest will influence their behavior (it means click or not). In 2018, Alibaba proposed a deep interest network (DIN) that adaptively determines the representation vector of user interest by considering the significance of a specific candidate advertisement’s previous behavior. In the context of e-commerce apps, it pays closer attention to user behavior. DIN models this process by concentrating on the manifestation of local activation interest for a specific ad. It pays attention to user behavior in the scene of e-commerce applications. DIN simulates this process by focusing on the expression of local activation interest for a given advertisement. DIN does not use the same vector to express the different interests of all users but adaptively calculates the vector of user interests by considering the relevance of historical behavior candidate advertisements.

The architecture of DIN (Zhou et al., 2018; Zhou et al., 2019) is shown in Fig. 1. A newly designed activation unit using a neural network was obtained, and the other structures are as same as the base model. In the traditional Attention mechanism, two-item embeddings such as u and v usually obtain the dot product uv or uWv directly, where W is a weight matrix of —u—x—v—. A new solution using feature combination proposed using the shop attribute of an ad to hit the shop list of the user’s historical behavior. If it is hit, it means that the historical user has had a direct behavior. User behavior id and frequency represent this combined feature; if there is no hit, the feature is empty. The diversity of behavior data reflects users’ various interests. A user’s click of an ad often originates from only a part of the user’s interests. In the NMT task, it is assumed that the importance of each word in each decoding process is different in a sentence. The attention network can be considered a mainly constructed pooling layer that learns to assign attention ratings to each word in a phrase based on data variety.

The activation units are applied as a weighted sum pool to the user behavior features to adaptively generate the user representation, where *vU* of a particular candidate advertisement is designated as A. The original user behavior embedding vector and the advertising embedding vector are two parts of the activation unit’s input; the other is the vector obtained by calculating the outer product of the two embedding vectors:

(2) \begin{eqnarray*}{v}_{u \left( A \right) }=~F \left( {v}_{A},~{e}_{1},{e}_{2},\ldots ,{e}_{H} \right) =~\sum _{j=1}^{H}a \left( {e}_{j},{v}_{A} \right) {e}_{j}=\sum _{j=1}^{H}{\omega }_{j}{e}_{j}.\end{eqnarray*}

**Figure 2:**
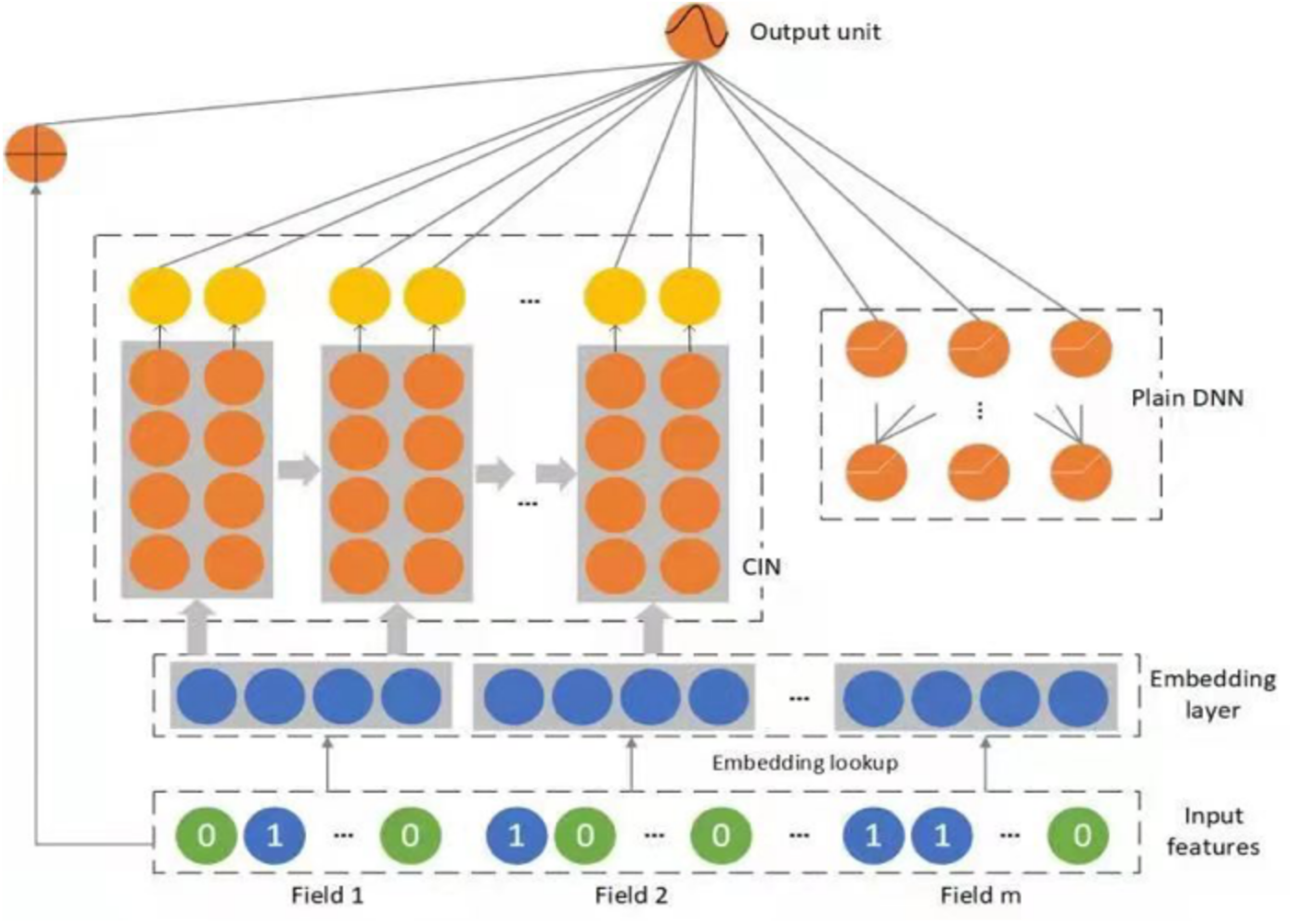
The proposed structure of DIN.

The list of embedding vectors of behaviors of user U with a length of H is *e*1, *e*2, *dots*, *eH*, and vA is the embedding vector of ad A. vU (A) fluctuates in this way across distinct adverts. A () is a feed-forward network with the activation weight as the output. Aside from the two input embedding vectors, a() adds the out product to feed into the following network, explicit information to help relevance modeling. The whole structure of DIN is shown in Fig. 2.

### xDeepFM

There are two high-order interactions: implicit high-order interactions and explicit high-order interactions.

#### Implicit high-order interactions

The implicit interactions (Liu et al., 2018) means the final results of the interactions are arbitrary, and there is currently no theory to demonstrate the maximum order of feature intersections that the methods—FNN, the deep part of Wide&Deep, and other CTR prediction models can learn. To learn high-order interactions, neural networks use a feed-forward neural network on the field embedding vector *e*. The formula for the procedure is as follows, where the layer depth is *k*, the activation function is *sigma*, and the bias is *b*∗.

(3) \begin{eqnarray*}{x}^{1}=~\sigma \left( {W}^{(1)}e+{b}^{1} \right) \end{eqnarray*}

(4) \begin{eqnarray*}{x}^{k}=~\sigma \left( {W}^{ \left( k \right) }{e}^{k-1}+{b}^{k} \right) .\end{eqnarray*}

#### Explicit high-order interactions

The direct interactions of Liu et al. (2018) are the interactions whose results are straightforward and can be derived. The most famous direct high-order interaction is the DCN, whose Cross network is an improved classical fully connected feed-forward network strategy. Moreover, the hidden layer is operated by the following operation:

(5) \begin{eqnarray*}{x}_{k}={{x}_{0}x}_{k-1}^{T}{\omega }_{k}+{b}_{k}+{x}_{k-1}\end{eqnarray*}

where *x*_*k*_ ∈ ℝ^*mD*^ is the output of the k-th layer, *ω*_*k*_ is the weight of the k-th layer, and *b*_*k*_ is the bias of the k-th layer. The Cross-layer can efficiently and explicitly learn high-order cross-features, but the problem is that a particular form limits their results, and feature crossovers are on a bitwise level rather than a vectorwise level. The unique structure of the Cross-layer allows it to display and automatically construct finite high-order feature fork multiplication.

**Figure 3:**
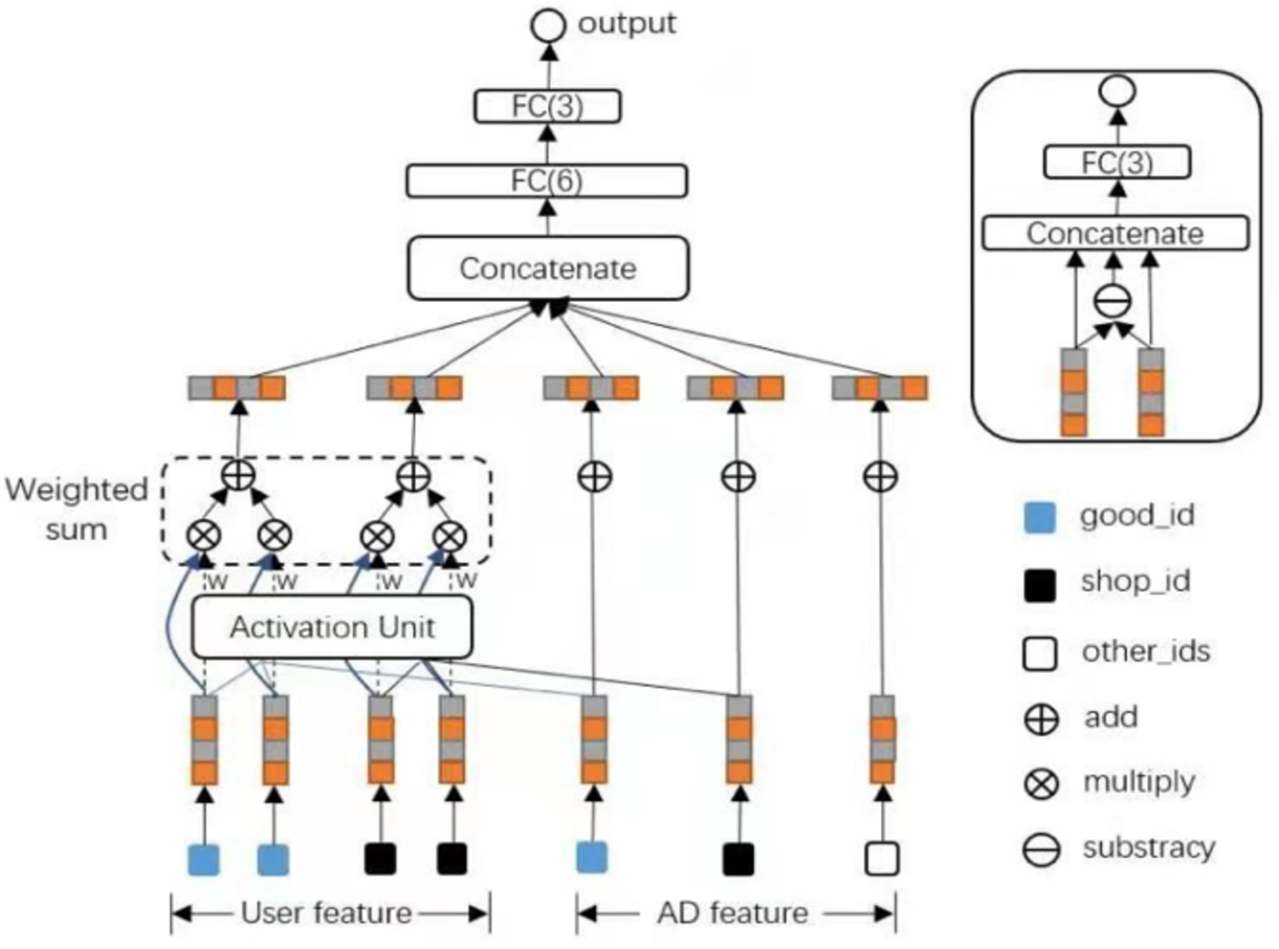
The proposed structure of xDeepFM.

DNN is a black-box model that implicitly interacts with high-level features. Therefore, the final result of DNN is arbitrary, and we cannot derive theoretically, nor can we obtain the maximum degree of feature interaction. Therefore, based on the idea of Deep&cross, xDeepFM was proposed in 2018 by Microsoft (Lian et al., 2018)—combining the classic plain DNN, a linear part, and a novel Compressed Interaction Network (CIN)—efficiently capturing feature interactions of bounded degrees. Unlike traditional deep neural networks that generate feature interactions implicitly at a bitwise level, xDeepFM proposed CIN, which aims to generate feature interactions at the vector level in a straightforward way. The whole structure of xDeepFM is shown in Fig. 3.

The structure is similar to Wide&Deep and DCN, and there are three parts in the xDeepFM: linear layer, CIN layer, and Deep layer. Due to this, xDeepFM has two advantages. At first, the interactions are applied at both vectorwise and bitwise levels. Secondly, it includes both low-order and high-order feature interactions. High-level feature interaction is measured, and the complexity of the network does not increase exponentially with the degree of interaction. The resulting output is as follows:

(6) \begin{eqnarray*}\hat {y}=o \left( {w}_{linear}^{T}a+{w}_{dnn}^{T}{x}_{dnn}^{k}+{w}_{cin}^{T}{p}^{+}+b \right) \end{eqnarray*}

where *o* is the sigmoid function; *a* are the raw features; ${x}_{dnn}^{k}$ and *p*^+^ are the outputs of the plain DNN and CIN, respectively; and *W*∗ and *b* are learnable parameters.

The loss function is the negative log loss, which is the same as DIN’s loss.

(7) \begin{eqnarray*}L=- \frac{1}{N} \sum _{ \left( x,y \right) \mathrm{\in }s} \left( ylogp \left( x \right) + \left( 1-y \right) log \left( 1-p \left( x \right) \right) \right) .\end{eqnarray*}

With *x* as the network’s input and *yin*0, 1 as the label, *S* is the training set of size N and *p*(*x*) is the network’s output, reflecting the projected probability of sample *x* being clicked. Furthermore, the goal of optimization is to reduce the following objective function to the smallest possible value:

(8) \begin{eqnarray*}J=L+{\lambda }_{\ast } \left\vert \left\vert 0 \right\vert \right\vert \end{eqnarray*}

where *λ*_∗_ represents the regularization term and *λ*_∗_ denotes the parameters, including those in the linear, CIN, and DNN parts.

The essential point of xDeepFM is the CIN part. Cross-layer is the most refined grain with a single bit embedded in the vector, while FM is the finest-grained learning correlation, vectorwise. The motivation of xDeepFM is to introduce FM’s idea of vectorwise into the Cross-layer. The input of CIN is from the embedding layer. If there are field, and the dimension of every field’s embedding vector is D, the input will be a matrix *X*^0^ ∈*R*^*m*∗*D*^ and the k-th vector is calculated by this process:

(9) \begin{eqnarray*}{w}_{h,\ast }^{k}=\sum _{i=1}^{{H}_{k-1}}\sum _{j=1}^{m}{W}_{ij}^{k,h} \left( {X}_{i,\ast }^{k-1}\mathrm{ \circ }{X}_{j,\ast }^{0} \right) \mathrm{ \in }{R}^{\mathrm{1}\ast D},where~~1\leq h\leq {H}_{k}\end{eqnarray*}

where ${W}_{ij}^{k,h}\mathrm{ \in }{R}^{1-m\ast D}$ indicates the h-th vector weight matrix of a k-th layer. Note that *X*^*k*^ is derived *via* the interactions between *X*^*k*−1^ and *X*^0^, so the feature interaction is measured and the degree of interaction increases with the depth of the layer.

### Hybrid model

**Figure 4:**
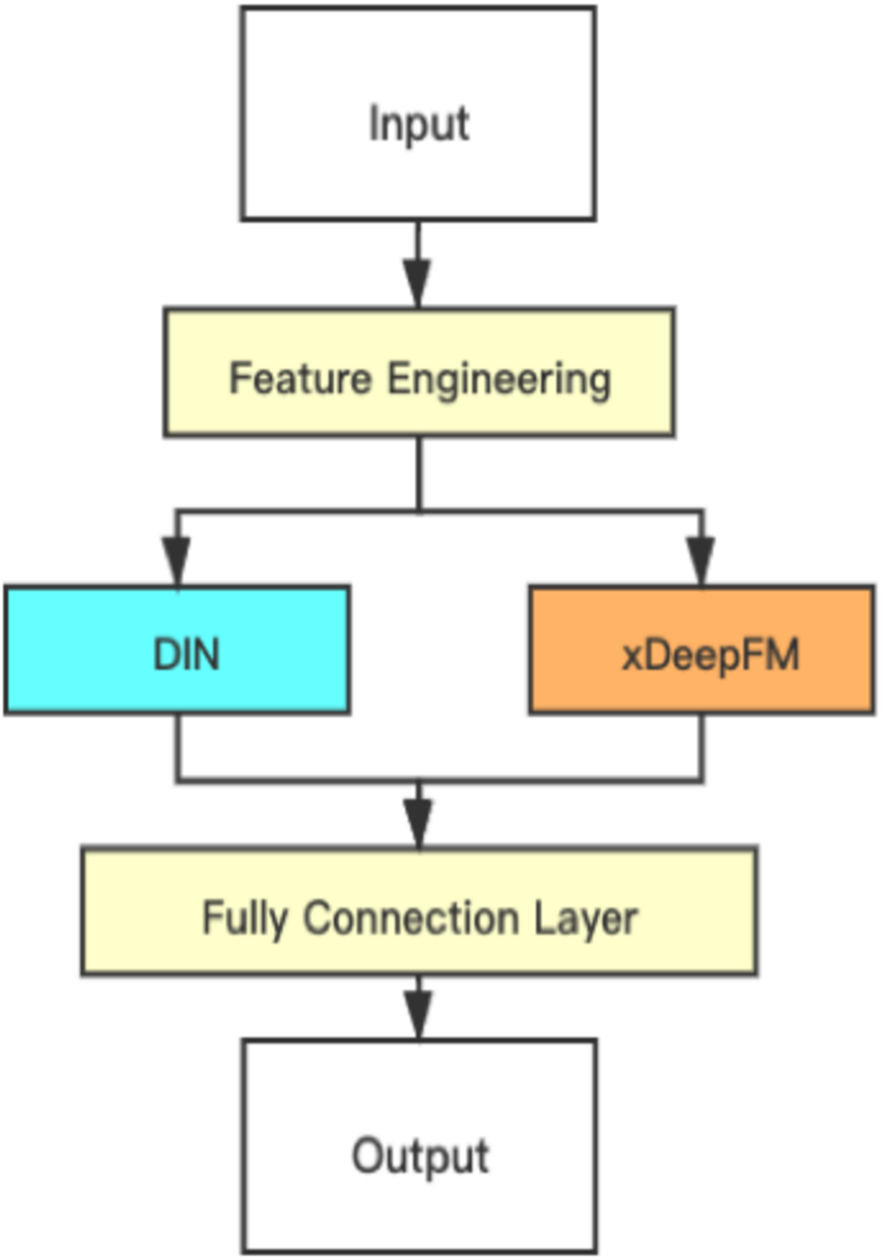
Structure of the proposed hybrid model.

As the DIN and xDeepFM focus on different parts, DIN uses an interest distribution to reflect users’ diverse interests and creates an attention-like network structure to locally activate associated interests based on the candidate ad, which effectively beats standard models. On the other hand, the xDeepFM focuses on combining implicit interactions and the direct interaction at a vector level, not a bit level. This paper proposed combining them and connecting a fully connected layer to obtain the final result. The structure of the proposed hybrid model is shown in Fig. 4.

The final result of the hybrid model is obtained with the following formula:

(10) \begin{eqnarray*}\hat {y}={W}_{DIN}^{T}{x}_{DIN}^{k}+{W}_{XdeepFM}^{T}{x}_{XdeepFM}^{T}+b.\end{eqnarray*}

The loss function we use is the focal loss, and it’s formula is shown as follows:

(11) \begin{eqnarray*}{L}_{fl}= \left\{ \begin{array}{@{}c@{}} \displaystyle -\aleph { \left( 1-{y}^{{^{\prime}}} \right) }^{\gamma }log{y}^{{^{\prime}}},~~y=1\\ \displaystyle -(1-\aleph ){{y}^{{^{\prime}}}}^{\gamma }log(1-{y}^{{^{\prime}}}),~~y=0. \end{array} \right. \end{eqnarray*}

Kaiming proposed the above-stated focal loss (Lin et al., 2017); essentially, the focal loss solves the imbalance and difficulty of classification in the classification problem. Focal loss is the improvement of the cross-entropy. Based on the original loss, it adds a parameter *γ* to decrease the loss of the easily classified sample. Therefore, it places a high value on the problematic and misclassified samples. Besides, it also introduced a balanced parameter ℵ to balance the uneven ratio of positive and negative samples. The objective of the proposed hybrid model is to minimize focal loss.

## Experiments

This section introduces the dataset and preprocessing. The metrics we chose to evaluate the models’ performance are accuracy and AUC–ROC. In addition, we use complex online example mining to improve the training effect. Finally, we compare the hybrid model with xDeepFM, DeepFM, DIN, and LightGBM on the metrics (accuracy and AUC–ROC).

### Data introduction

Alibaba provides the dataset from the Kaggle website, which indicates the click rate prediction regarding displayed Ads. In Table 1, we show components of Taobao’s dataset. The dataset used for the research is available at Sanagapati (2017). Similarly, Tables 2 and 3 represents Raw_sample and Ad_feature items and their descriptions. User_profile items, Behavior_log items and their description are given in Tables 4 and 5.

**Table 1:** Datasets used and their detailed descriptions.

Table	Description	Feature
raw_sample	The skeleton of raw training samples	User ID, Ad ID, nonclk, clk, timestamp
ad_feature	ad’s basic information	Ad ID, campaign ID, Cate ID, Brand
user_profile	user profile	User ID, age, gender, etc
raw_behavior_log	user behavior log	User ID, btag, cate , brand, timestamp

**Table 2:** Raw_sample items and their descriptions.

Field	Description
clk	1 for click, 0 for not click
noclk	1 for not click, 0 for click
pid	Scenario
addgroup_id	Add group ID (int)
time_stamp	time stamp (Bigint, 1494032110 stands for 2017-05-06 08:55:10)
User	User ID (int)

**Table 3:** Ad_feature items and their descriptions.

Field	Description
pri	item price
cus_id	advertiser ID
Brand	brand ID
cam_id	campaign ID
cat_id	kinds ID
ad_id	ad ID (int)

**Table 4:** User_profile items and their descriptions.

Field	Description
new_user_class_level	city level
Occupation	is college student 1—yes, 0—no?
shopping_level	shopping depth: 1—shallow user, 2—moderate user, 3—depth user
pvalue_level	consumption grade: 1—low, 2—mid, 3—high
age_level	age_level
final_gender_code	gender: 1 for male, 2 for female
cms_group_id	cms_group_id
cms_micro_id	Micro group ID
Userid	user ID (int)

**Table 5:** Behavior_log items and their descriptions.

Field	Description	
time_stamp	(Bigint, 1494032110)	
nick		
	User ID (int)	
btag	Types of behavior, including the following four	Buy (buy)
		Fav (favor)
		Cart (browse)
		Ipv (explanation)
brand	Brand id (int)	
cate	Category id (int)	

The original sample skeleton has 1140000 users from the website of Taobao for 8 days of ad display/click logs (26 million records).

All ads in raw_sample are covered in the basic information.

An item is identified by one of the ad IDs; an item belongs to a category and an item belongs to a brand. The user_profile contains the basic information of 1060000 users in the raw sample.

The behavior_log displays the shopping behavior of all users in the raw_sample over the course of 22 days.

We can find numerous duplicate records if we utilize the user ID and timestamp as the primary key. Different forms of data behave differently in different departments and, when packed together, there is a tiny variance (*i.e.,* the identical two timestamps may be two different times with relatively minor differences).

### Preprocessing

**Figure 5:**
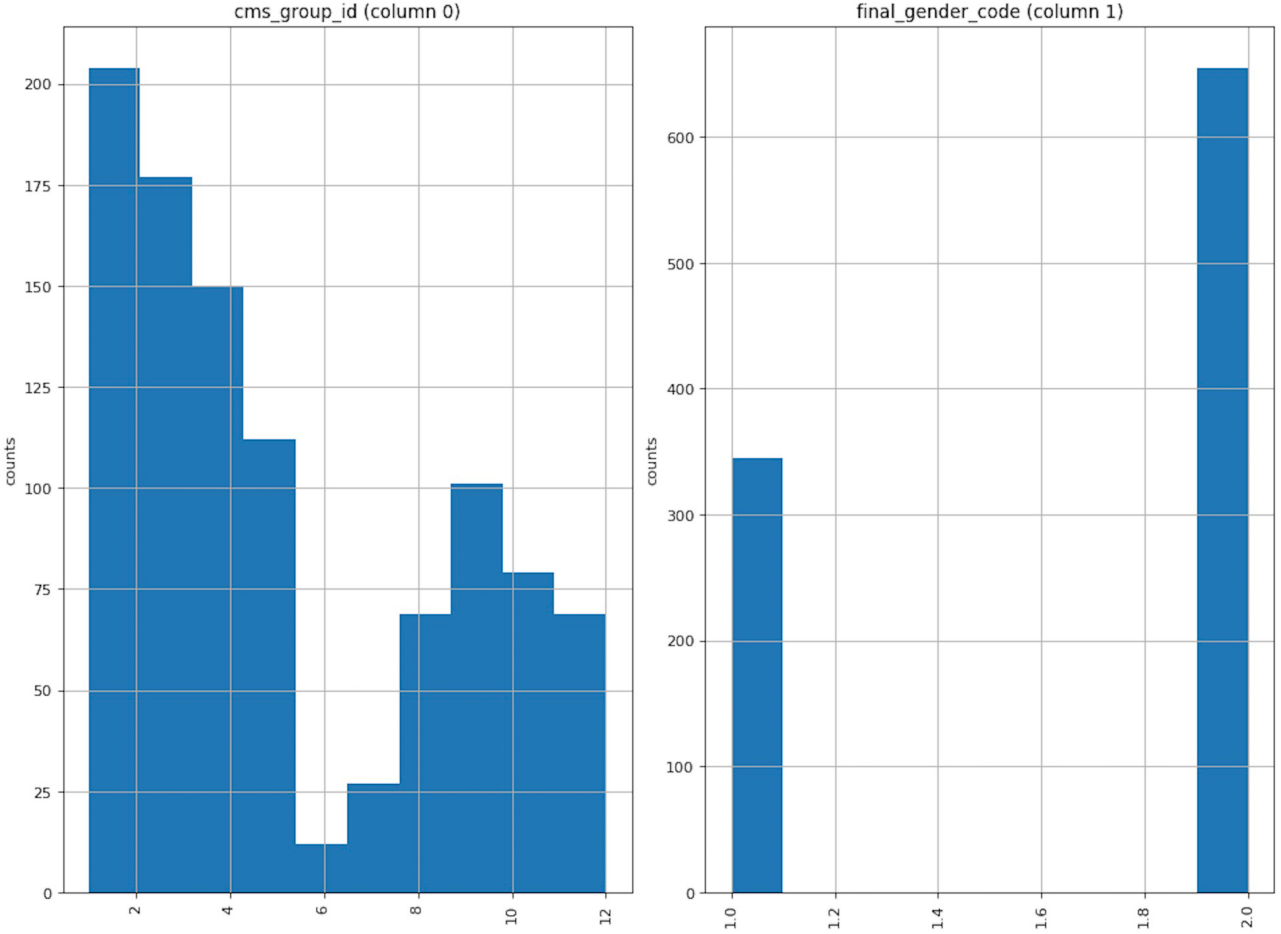
Data distribution.

To describe the data distribution, we choose the cms_group_id and final_gender_code as examples, drawing the distribution in Fig. 5. The left figure shows the cms_group_id; it is evident that the group’s quantity of the group_id in 6 is small while the group_id in 1 is vast. The correct figure shows the final_gender_code; from the raw dataset, we know the number of class 1 is less than that of class 2, which means there are more female users than male users. This is consistent with popular perception.

**Figure 6:**
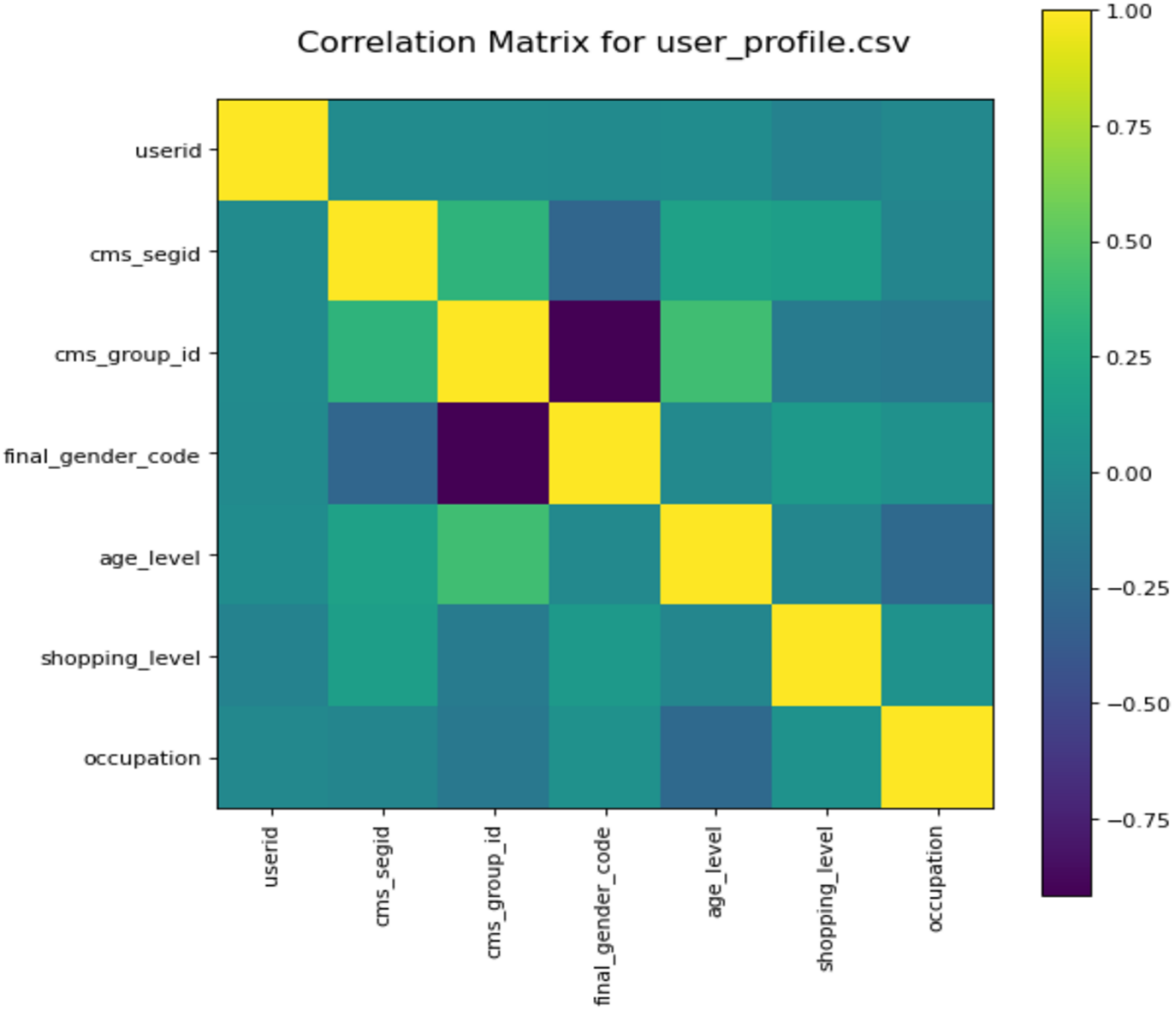
Co-relation metrics obtained for user profiles.

To better understand the used data, we also analyze the correlation of different user profiles. We draw the correlation matrix (Steiger, 1980) *via* computing Pearson’s correlation coefficient for each feature in the user profile. Figure 6 shows the correlation matrix. The closer the color is too yellow, the stronger the correlation between the two features. From Fig. 6, we can see the correlation of different features. For example, the relationship between features cms_group_id and final_gender_code is a negative coefficient, and the relationship between user and occupation is an irrelevance.

### Competitors

(1) xDeepFM: As the proposed model is a hybrid model combined with xDeepFM and the Deep Interest Network, it is necessary to compare its performance with the individual model.

(2) Deep Interest Network: We chose the Deep Interest Network for the same reason as xDeepFM.

(3) DeepFM: Compared with xDeepFM, which is a generalization of DeepFM by learning the linear regression weights for the FM layer, DeepFM is also chosen for the competitions.

(4) LightGBM (Ke et al., 2017): LightGBM was proposed by MSRA in 2019 using Exclusive Feature Bundling (EFB) to decrease features and has become a popular method in CTR prediction tasks.

### Evaluation metrics

**Figure 7:**
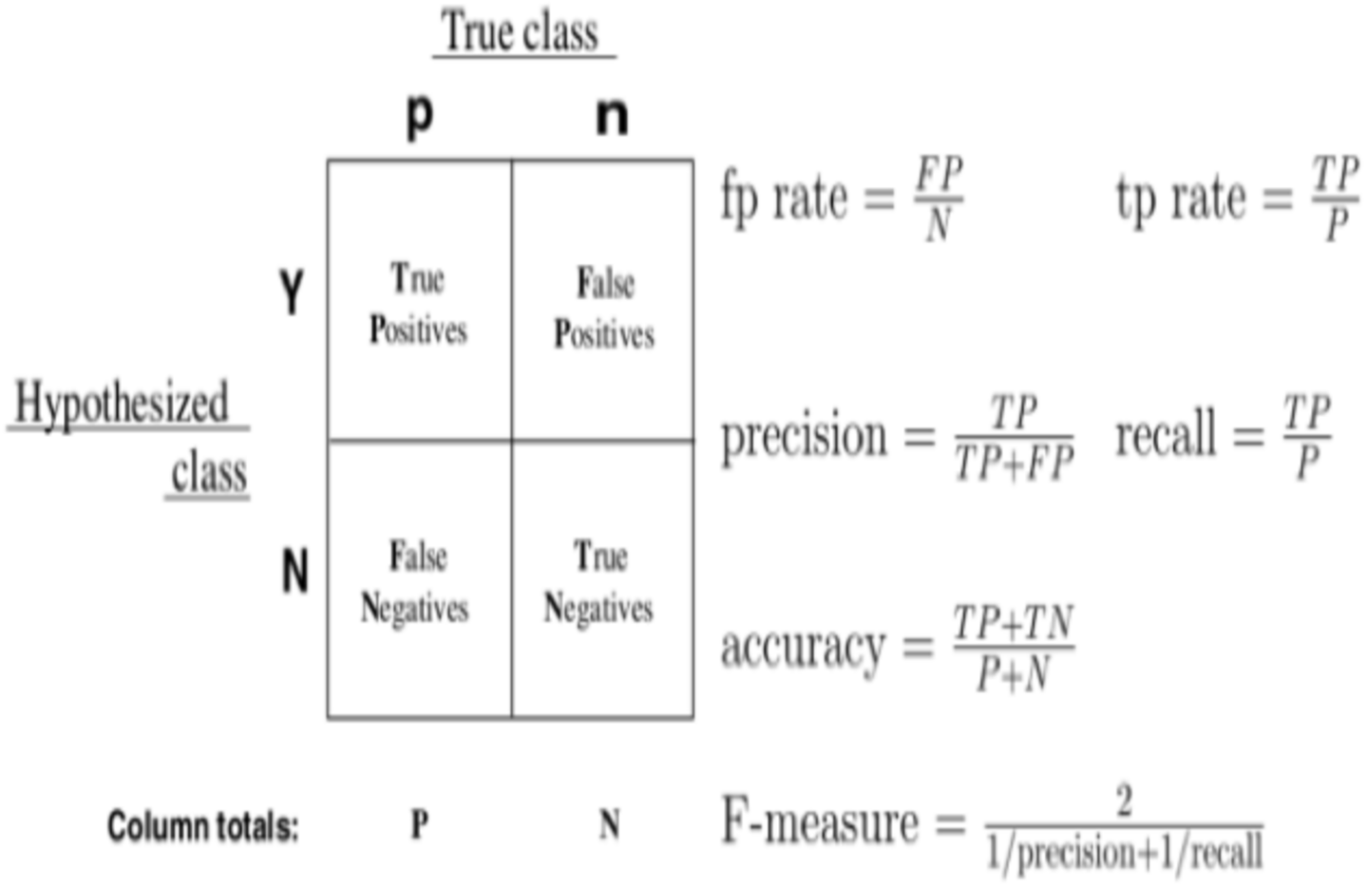
Graphical representation of confusion metrics’ descriptions and formulas.

As evaluation metrics, we employed accuracy and AUC–ROC (Davis & Goadrich, 2006). We commonly classify the result into four conditions for a classifier, as shown in the diagram: TP, TN, FP, and FN. The CTR methodology can also be evaluated using precision and recall. However, the outcomes of the proposed method are highly dependent on the test data sample, with slightly different test datasets yielding significantly different findings. A graphical representation of the confusion metrics’ descriptions and formulas are given in Fig. 7.

We use the *accuracy* = *TP* + *TN*/*P* + *N* (Narkhede, 2018) to find out how accurate the CTR prediction is; AUC also assesses the quality of the order by ranking all the ads based on their anticipated CTR. A change in user-weighted AUC was recently employed, which analyses the user’s internal ordering validity using the mean user’s AUC and has proven more significant to the digital advertising system’s online performance. AUC tolerates changes in sample proportions to a certain extent. AUC generally falls in the range of 0.6−0.85. The mathematical formula for calculating AUC is given below:

(12) \begin{eqnarray*}AUC= \frac{\sum _{i=1}^{n}#impressio{n}_{i}\mathrm{ \times }AU{C}_{i}}{\sum _{i=1}^{n}#impressio{n}_{i}} \end{eqnarray*}

where *n* is the number of users, ${\mathop{\sum }\nolimits }_{i=1}^{n}#impressio{n}_{i}~$ and *AUC*_*i*_ are the number of impressions, and AUC corresponds to the i-th user.

The AUC–ROC (Gu, Ghosal & Roy, 2008; Shrivastava, Gupta & Girshick, 2016) meter is utilized as the metric in the suggested method to quantify the accuracy of CTR estimation. We employ the ROC curve, which has the advantage of remaining unaltered when the distribution of positive and negative samples in the test set changes. There is frequently a class imbalance phenomenon in actual datasets. There are far fewer positive examples than negative samples and vice versa. Additionally, the proportion of positive and negative instances in the testing dataset may alter with time. The area under this curve is calculated as the AUC value; the higher the AUC value, the more accurate the forecast.

We apply the online complicated example mining (OHEM) (An et al., 2010) algorithm throughout the training process to boost the model’s performance. In deep learning, OHEM is a bootstrap application. The algorithm is a straightforward adaptation of the SGD. The training set is sampled from a nonstationary, uneven distribution. The troublesome cases are retrained after each batch. The specific method first calculates the loss for all samples using DL; then, using the loss as the standard of a complex example to choose the rigid model, it finally forms a batch for training.

Table 6 shows five different CTR prediction methods with the same training dataset with their AUC–ROC and accuracy. The higher AUC–ROC and accuracy are, the better performance the model will obtain. The proposed hybrid model’s AUC–ROC is 0.661, 0.009, 0.014, and 0.008–−0.018 higher than xDeepFM, DeepFM, Deep Interest Network, and LightGBM. Simultaneously, the accuracy of the hybrid model is also the highest among these models, and its score is 0.956. Both AUC–ROC and accuracy show that the performance of the hybrid model is better than other models, which is an optimal choice for CTR prediction.

**Figure 8:**
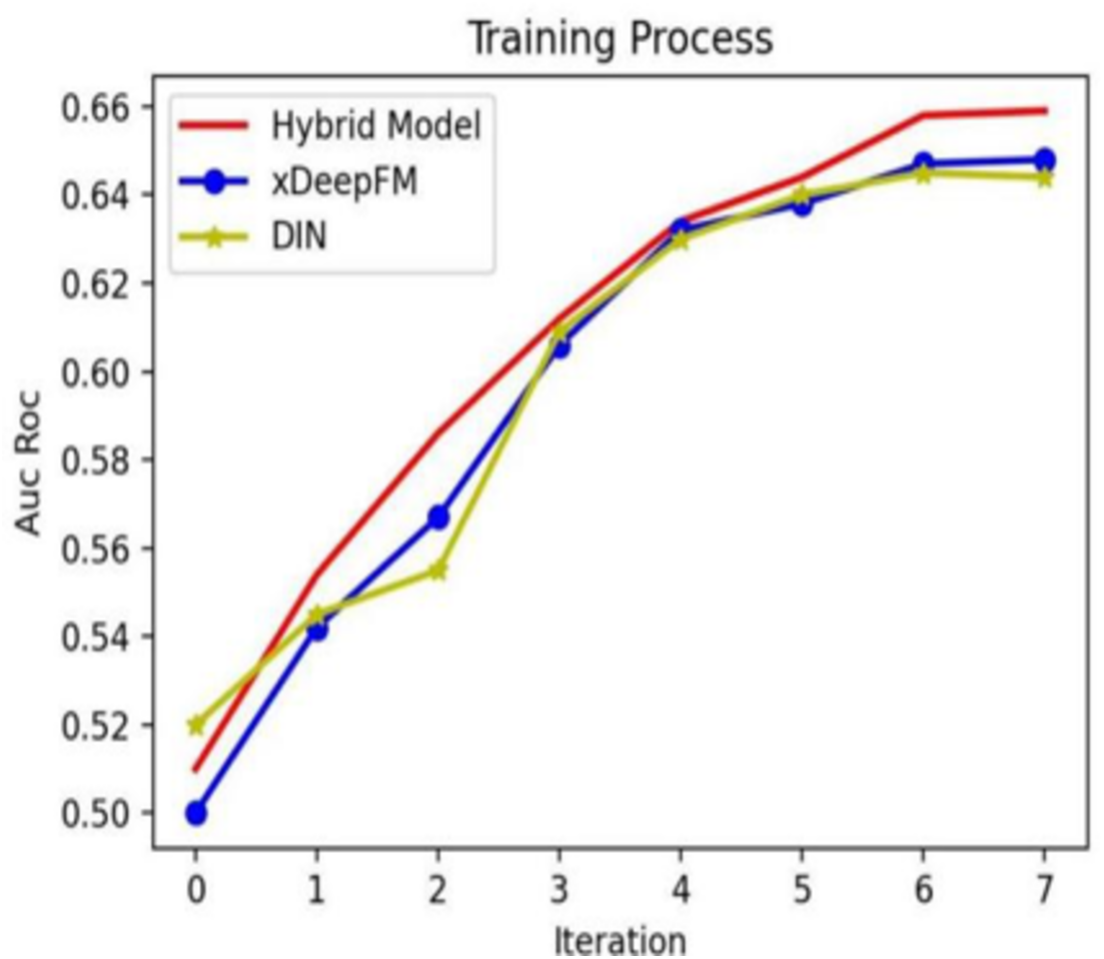
Different models’ training processes on different Epoch configurations.

In addition, to compare the hybrid model with the individual models, we show the training process in Fig. 8. From Fig. 8, it is evident that with the increasing iteration, the AUC–ROC of three methods all increase, and the hybrid model’s performance is the best.

## Conclusion

In recent years, CTR prediction has been a primary task in the advertising system. This article focuses on a series of traditional CTR estimation algorithms, such as the Logistic regression algorithm and the latest popular deep neural network methods, for example, DIN, DeepFM, and other related variants for advertising the CTR prediction strategy. The proposed hybrid model is based on DIN and xDeepFM to obtain better CTR prediction performance. The core of DIN and xDeepFM are attention and feature intersection, respectively. DIN follows a local activation unit to adaptively learn the expression of user interest from the historical behavior of specific advertisements.

**Table 6:** Performance evaluation of different models.

Models	AUC-ROC	Accuracy
Hybrid Model	0.661	0.956
xDeepFM	0.652	0.947
DeepFM	0.647	0.930
Deep Interest Network	0.653	0.945
LightGBM	0.643	0.931

On the other hand, xDeepFM acquaints an integral part of the Compressed Interactions Network (CIN), aiming to generate feature interactions at the vector level implicitly. Furthermore, the proposed model merges a CIN, a classic DNN, and a linear part into a unified model; the new model is xDeepFM. The hybrid model and the end-to-end model are integrated through the parallel model of the multilayer perceptron, and their output is fed to the multilayer perceptron. The outcomes show that the proposed hybrid model obtained improved performance compared with the other models. In the future, we aim to test metaheuristic algorithms to optimize the hyperparameters of the proposed approach to enhance accuracy.

## Supplemental Information

10.7717/peerj-cs.716/supp-1 Supplemental Information 1 Computer Code for the proposed structure of DIN Proposed Model Code

## Additional Information and Declarations

### Data Availability

The following information was supplied regarding data availability: The code is available in the Supplemental File and the raw data is available at Kaggle: https://www.kaggle.com/pavansanagapati/ad-displayclick-data-on-taobaocom.
